# From Khoi-San indigenous knowledge to bioengineered CeO_2_ nanocrystals to exceptional UV-blocking green nanocosmetics

**DOI:** 10.1038/s41598-022-06828-x

**Published:** 2022-03-02

**Authors:** N. Ditlopo, N. Sintwa, S. Khamlich, E. Manikandan, K. Gnanasekaran, M. Henini, A. Gibaud, A. Krief, M. Maaza

**Affiliations:** 1 College of Graduate Studies, UNESCO-UNISA Africa Chair in Nanosciences-Nanotechnology, Muckleneuk Ridge, PO Box 392, Pretoria, South Africa; 2 Nanosciences African Network (NANOAFNET), iThemba LABS-National Research Foundation, 1 Old Faure Road, PO Box 722, Somerset West, 7129 Western Cape South Africa; 3 Physics Deptartment, TUCAS Campus, Thiruvalluvar University Serkadu, Vellore, 632115 India; 4 P.G. and Research Physics Department, A M Jain College, University of Madras, Meenambakkam, Tamil Nadu 600114 India; 5 Physics Department, University of Nottingham, Nottingham, UK; 6 IMMM, UMR 6283 CNRS, University of Le Maine, Bd O. Messiaen, 72085 Le Mans Cedex 09, France; 7 International Organization for Chemistry in Development, Liege, Belgium

**Keywords:** Biophysics, Materials science, Nanoscience and technology

## Abstract

Single phase CeO_2_ nanocrystals were bio-synthesized using Hoodia *gordonii* natural extract as an effective chelating agent. The nanocrystals with an average diameter of 〈Ø〉 ~ 5–26 nm with 4^+^ electronic valence of Ce displayed a remarkable UV selectivity and an exceptional photostability. The diffuse reflectivity profile of such CeO_2_ exhibited a unique UV selectivity, in a form of a Heaviside function-like type profile in the solar spectrum. While the UV reflectivity is significantly low; within the range of 0.7%, it reaches 63% in the VIS and NIR. Their relative Reactive Oxygen Species (ROS) production was found to be < 1 within a wide range of concentration (0.5–1000 μg/ml). This exceptional photostability conjugated to a sound UV selectivity opens a potential horizon to a novel family of green nano-cosmetics by green nano-processing.

## Introduction

**Figure 1:**
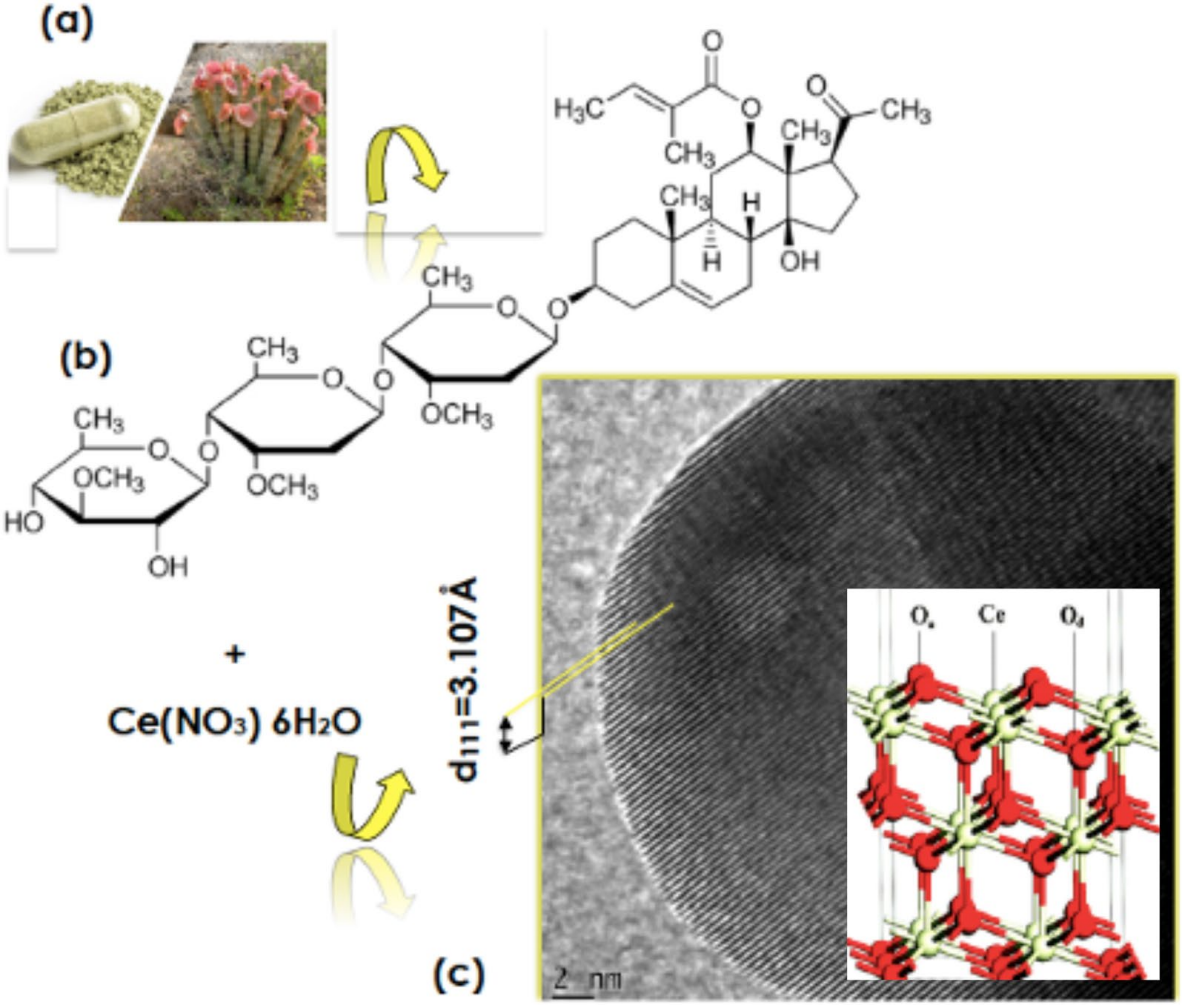
(**a**) Hoodia *gordonii* plant and in a powdered form, (**b**) chemical structure of the most active biocompound known as P57 (12-O-Trigloyl-3β,12β,14β-pregn-5-en-20-one3-O-β-D-thevetopyranosyl-(1 → 4)-β-D-cymaropyranosyl-(1 → 4)-β-d-cymaro-pyranoside), (**c**) typical HRTEM scan of a biosynthesized CeO_2_ nanocrystal.

Known to the Khoi and the San communities of Southern Africa, the Hoodia *gordonii* first, described by botanist R. Sweet in Hortus Britannicus in 1830^1^, is a succulent plant from the Apocynaceae family (Fig. 1a). It is indigenous to South Africa and Namibia. It exhibits a significant tolerance to harsh conditions and grows in extreme environments of heat (40 °C) to low temperatures (− 3 °C). Within the modern obesity health problem, this indigenous plant has attracted a noteworthy pharmaceutical interest as per its proven traditional usage by the Khoi-San community as an appetite and thirst effective suppressant. Several bioactive compounds have been identified and isolated from its natural extract. The major bioactive compound consists of a group of glycosides, especially the one referred to as steroidal glycoside and its collective analogs such as the so called P57 which chemical structure is given in Fig. 1b. Considering the potential aldehyde components within its bioactive compounds, its natural extract is likely to be effective as an efficient chelating agent for bio-engineering nanoscaled oxides^2,3^ such as Cerium oxide (CeO_2_) (Fig. 1c).

The focus on such a multifunctional Cerium oxide^4–9^ in this contribution is geared towards investigating its UV filtering selectivity in the perspective of designing a novel family of green nanocosmetics by an entirely green chemistry and green nano-processing. CeO_2_’s wide band gap (E_g_: 3.0–3.6 eV) and its relatively high refractive index (n: 2.2–2.8) substantiate its application in UV radiations protection sunscreens^10–13^ minimizing the risk of melanoma, sunburns and premature ageing as well as skin malignancy and related tumors.

**Figure 2:**
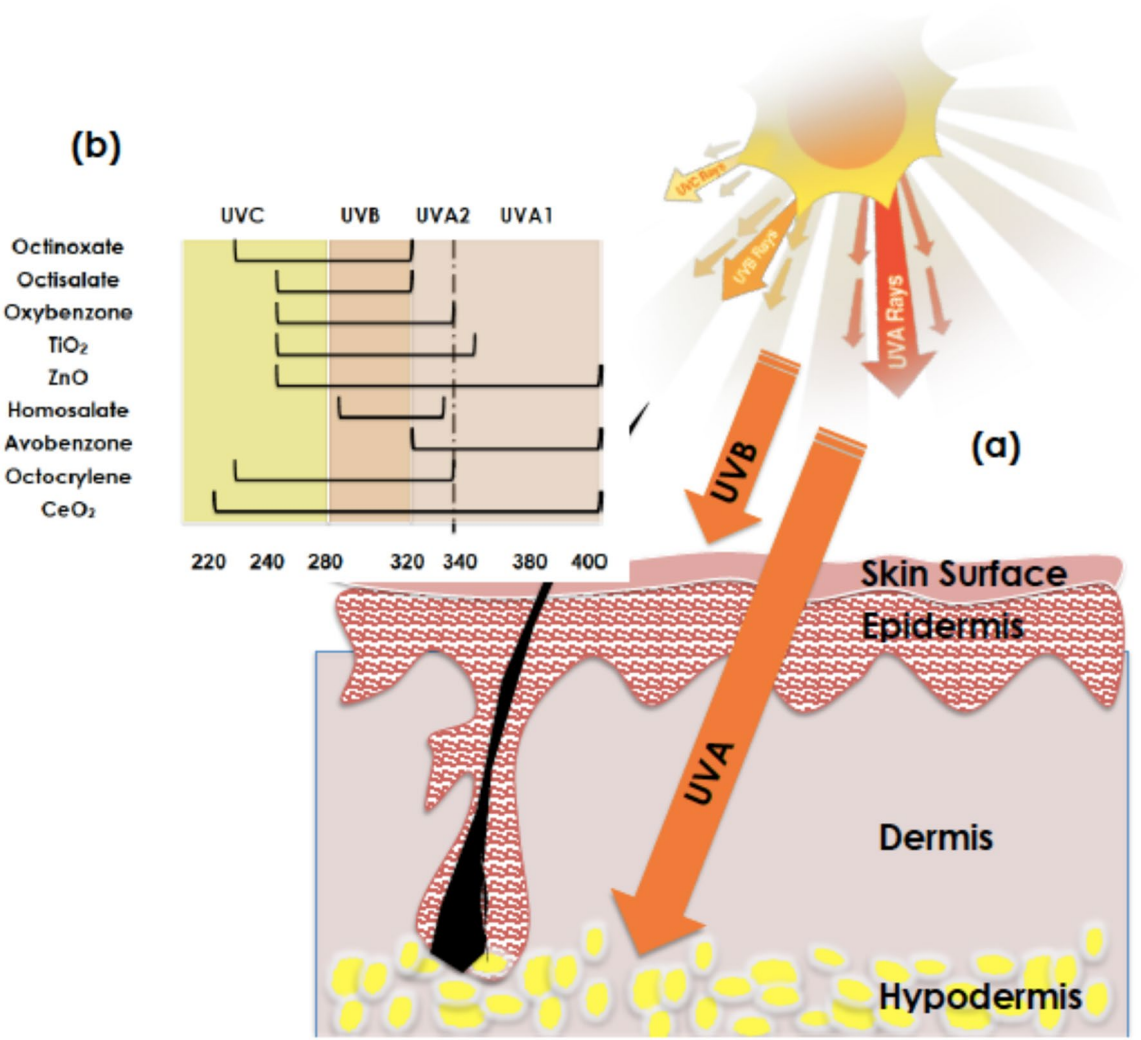
(**a**) Depth penetration of the various UV solar radiations within a human skin, (**b**) UV protection window of the current biosynthesized CeO_2_ compared to those standard compounds (organics) and traditional nanoscaled oxides ZnO and TiO_2_.

Within this contribution, it is reported that biosynthesized CeO_2_ nanocrystals using Hoodia *gordonii* natural extract as a chelating agent exhibit an exceptional UV selectivity in the 3 UV range quasi-similar to a quasi-perfect UV selectivity response with a negligible ROS factor. With such a UV selectivity and photostability, the current bio-synthesized CeO_2_ nano-powder would, likely, open the horizon for a novel green UV blocking biocompatible nano-sunscreen family. Indeed, as schematically reported in Fig. 2a, the solar UV radiations consists of 3 major components UVA (320–400 nm, deep penetrating rays that contribute to premature skin aging and wrinkling), UVB(280–320 nm, less penetrating rays that induce skin reddening and sunburn) and UVC(100–280 nm, most dangerous radiations but normally blocked by the earth’s O_3_ layer, O_2_, and H_2_O vapor present in the upper atmosphere of Earth). Figure 2b summarizes the various organic and inorganic major compounds available in the market as UV blocking sunscreens^13^, namely; Octinoxate, Octisalate, Oxybenzone, Homosalate, Avobenzone, Octocrylene, TiO_2_, ZnO.

As it will be sustained throughout the manuscript, the novelty and the originality of this contribution lie within the following major aspects:

(i) it is the first time within the scientific literature that Hoodia gordonii was used for the biosynthesis of nanoscaled CeO_2_ in addition to the usage of hoodia gordonii as an appetite and thirst suppressant,
(ii) it is the first time that such a biosynthesized nano-CeO_2_ exhibit a combined effective UV strong optical absorption, and a sound effectiveness against 2 major bacteria species, in addition to its minute ROS production relatively to standard oxides including TiO_2_ and ZnO. This very low ROS production is of importance in minimizing the DNA induced damages,
(iii) Last but not least, this contribution sustains the importance of the indigenous knowledge and the valorization of anthropological aspects via scientifically supported studies.

## Experiments and results

### Biosynthesis methodology

For the biosynthesis typical phase, ground powder of Hoodia *gordonii* from Dischem South Africa was used. All used additional chemical reagents were purchased from Sigma-Aldrich. They were all of analytical grade, hence used without any additional purification. In a typical procedure, 10.0 g of clean Hoodia *gordonii* powder was weighed and mixed to 400 ml of deionized H_2_O. The solution was kept at room temperature for about ~ 2 h. Thereafter, the obtained solution was filtered twice using Whatman filters to eliminate any residual solids. Following such a phase, 2.0 g of Ce(NO_3_)_3_ 6H_2_O was added to 100 ml of the filtered extract solution. The solution was then mixed under a thorough magnetic stirring and heated for ~ 2 h at about 48–50 °C. A solid precipitate was observed upon cooling. This deposit is likely to be CeOς or/and Ce(OH)ς or mixture of both. The solid deposit was purified by 2 repeated centrifugations at ~ 10,000 rpm for 10 min each. It was then dried in oven set at ~ 100 °C and thereafter annealed at various temperatures; 100, 200, 300, 400, 500, 700 °C for 2 h under air using a standard tubular furnace in view of crystalizing the likely CeOς/ Ce (OH)ς .

### Morphology and crystalline atomic structure

**Figure 3:**
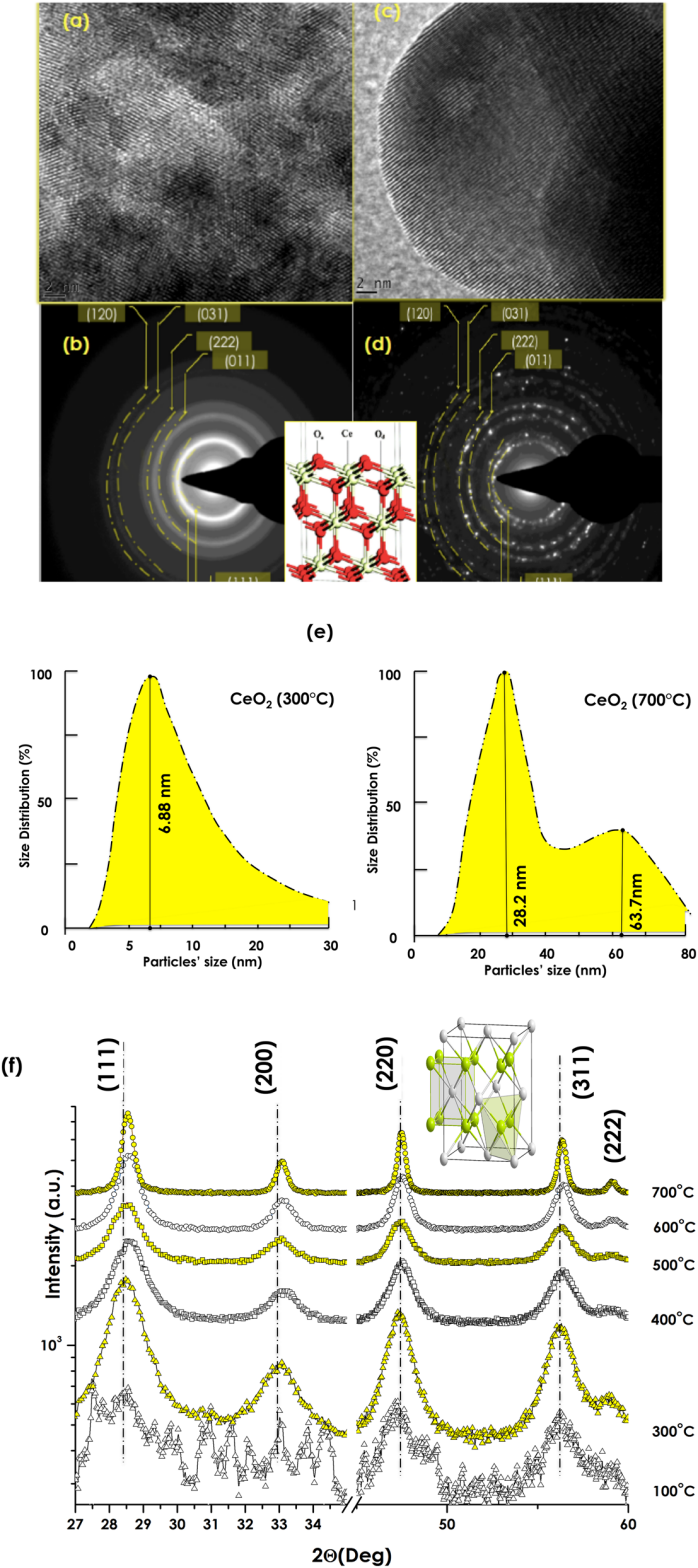
HRTEM of the CeO_2_ powder and annealed at ~ 300 °C (**a**) and  ~ 700 °C (**c**) respectively and their corresponding SAED (**b**,**d**), DLS size distribution (**e**) and the XRD spectra of the various samples (**f**).

Figure 3a–d report characteristic HRTEM micrographs and SAED patterns obtained on the CeOς/ Ce(OH)ς powder annealed at ~ 300 °C and  ~ 700 °C respectively. in both cases, the particles are nano-scaled. The corresponding average size 〈Ø〉 of the particles (derived from a J-Image data treatment of the TEM images) ranges within 4.68–8.83 nm and 5.81–71.3 nm for the samples annealed at 300 °C and 700 °C respectively. Light scattering size analysis of Fig. 3e suggests a single mode size distribution for the annealed sample at 300 °C with likely an average size-centered at about 6.9 nm. It is however required to be cautious with such a value as we are at the limit of the DLS methodology. The annealed sample at 700 °C seems exhibiting a bimodal size distribution centered at 28.2 and 63.7 nm respectively**.** As one notices in Fig. 3a, the 300 °C annealed nano-powdered sample consists of agglomerates of various nano-crystals with different crystalline orientations as reflected by the polycrystalline nature of the SAED pattern of Fig. 3c. In contrast, for the ~ 700 °C annealed sample, the nanocrystals are larger but still in the nanoscale while significantly crystalline (Fig. 3b) in agreement with the spot type electron diffraction pattern of Fig. 3d. In both cases, the indexation of the annular and singular diffraction patterns is in agreement with the face centered cubic phase of CeO_2_ (JCPDS 34–0394) corresponding to an average lattice constant of 〈a〉 = 3.865 Å which is the most reflecting reticular plans’distance d_111_ = 3.107 Å. More accurately, this later crystallographic phase consists of a cubic fluorite-type oxide in which each Ce site is surrounded by 8 O sites in an fcc arrangement while each O site occupies a tetrahedron Ce site. This seems to be in agreement with the X-Rays diffraction investigations of Fig. 3f. The following Bragg diffractions peaks are observed; (111), (220), (220), (311) as well as (222). Such a diffraction pattern fits with the single-phase Fluorite of polycrystalline CeO_2_. However, It is worth noting that each and all Bragg peaks’ angular position seem to shift towards higher angles relatively to those of the 300 °C annealed sample. Such an angular shift is relatively small of the order of 4.36 10^–3^ rad between the 300 °C (Smaller nanocrystals) and 700 °C (Larger nanocrystals). Hence all reticular atomic diffraction plans of the smaller nanocrystals especially will likely be submitted to a compression comparatively to the bulk. Such a relative compression is Δd/d = (cos Θ/sin Θ)ΔΘ. In the case of the nanocrystals of the CeO_2_ sample annealed at 300 °C, such a relative contraction Δd/d variation is of the order 6.014 10^–3^ induced likely by surface tension phenomena as it is the case in nanocrystals in general.

### Elemental analysis and surface coordination

**Figure 4:**
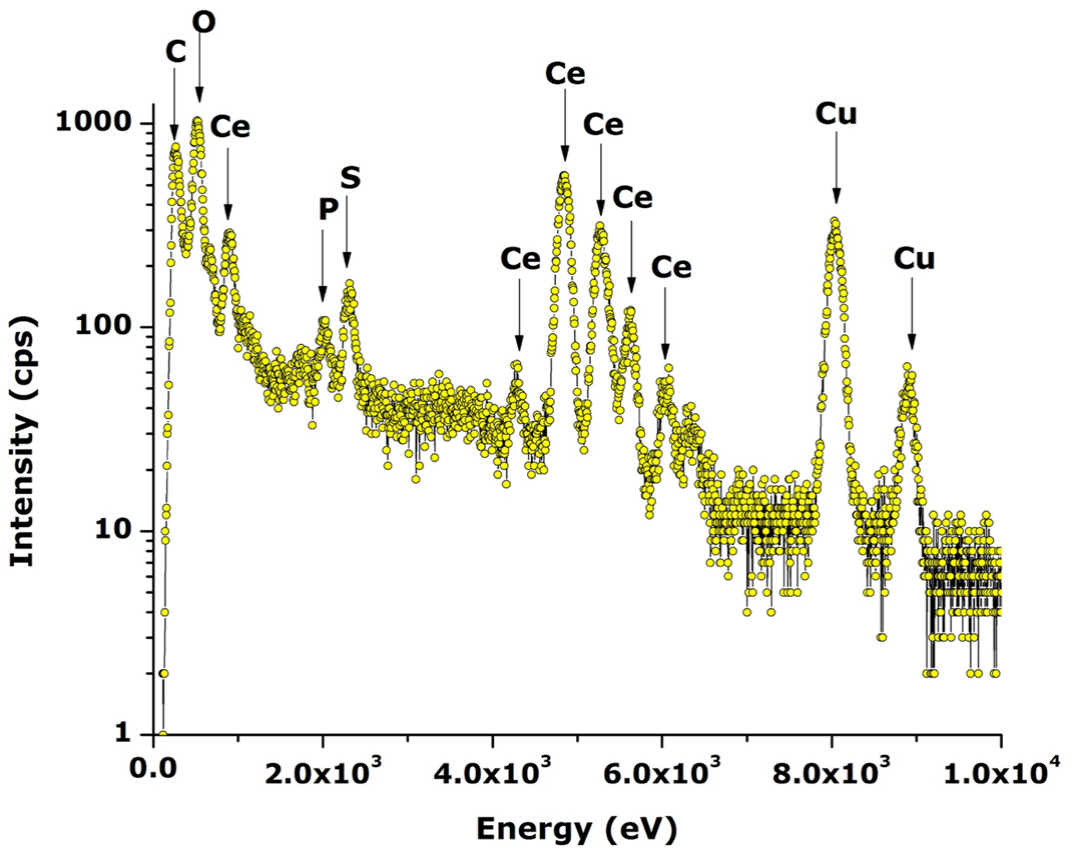
Representative EDS spectrum of CeO_2_ nano-powder annealed at ~ 700 °C.

Figure 4 shows a representative EDS elemental analysis spectrum in logarithmic intensity to put in evidence any potential contamination during the biosynthesis phase. As one can notice, excluding the carbon originating from the Carbon coated grid, there are no other elements observed except Cerium (Several peaks in relation to the f-electrons nature of Ce and Oxygen in addition to Sulfur and Potassium. These later ones, i.e. sulfur (S) and phophorus (P) originate likely from the natural extract’s organic compounds as it was observed previously in the case of several biosynthesis of several nan-oxides^2,14^.However, it is to be highlighted that the limit of detection of EDS being of the order of 10%, other contaminants can not be excluded if their concentration is below the 10% limit.

Likewise, the XPS studies of the annealed CeO_2_ nanoparticles were performed to investigate precisely the oxidation state of Ce and O (Fig. S1). The binding energy of CeO_2_ nanoparticles exhibited several bands with the main ones centered at about 882.2, 888.5, 897.7 and 916.3 eV corresponding to the Ce^4+^ 3d3/2 and Ce^4+^ 3d5/2 in addition to the O1s peak at 531 eV. The XPS observations are consistent with the reported values in the literature^14–18^. Hence, one can conclude that Ce is likely to be in 4 + valence state.

### UV Optical selectivity

**Figure 5:**
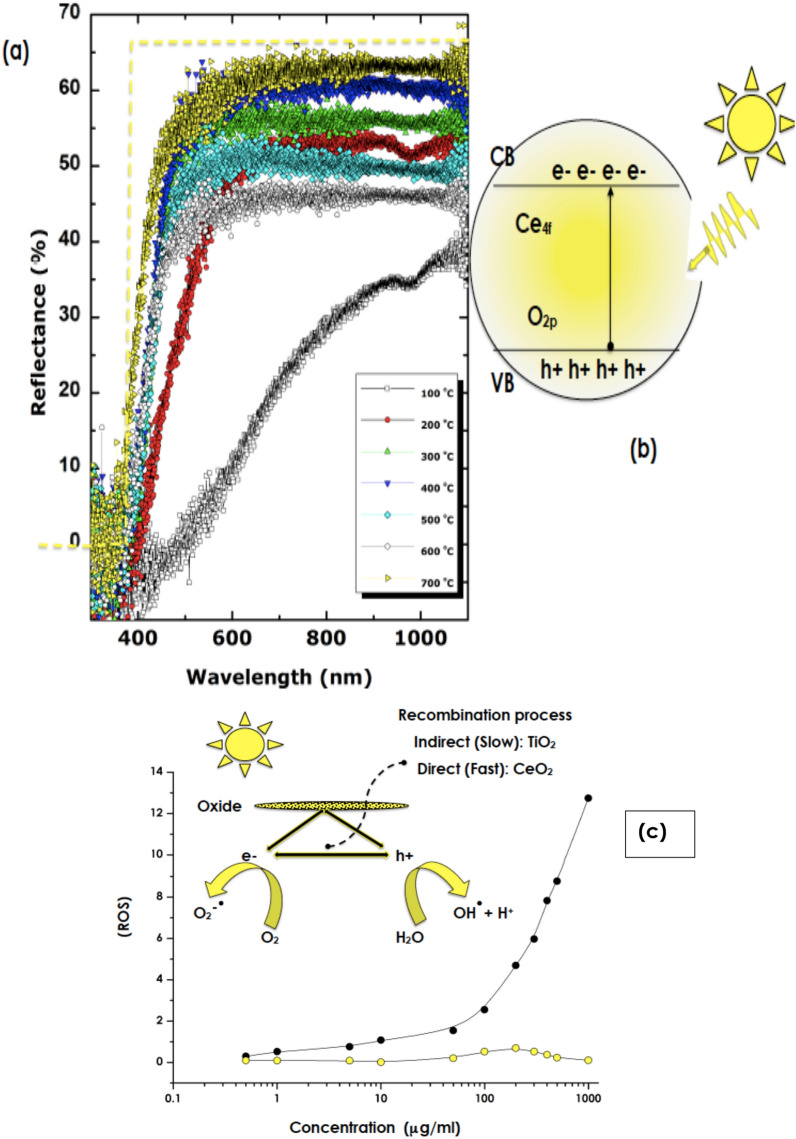
(**a**) Diffuse Reflectivity of the CeO_2_ annealed at various temperatures within the range of 100–700 °C, (**b**) band structure and the potential O 2p2 − to Ce 4f. 4 + transitions, (**c**) ROS production of the 700 °C annealed CeO_2_ (yellow circles) and standard nanoTiO_2_ (black closed circles), and inset (**b**) schematic representation of the e–h creation/recombination in TiO_2_ and CeO_2_.

Figure 5a reports the diffuse Reflectivity of the nanoscaled CeO_2_ pressed powder samples annealed at various temperatures. One can distinguish 2 major trends. The sample annealed at 100 °C and those above exhibit stringently different spectral variations. While the diffuse reflectance varies approximately in a linear way with the wavelength for the sample annealed at 100 °C, those above exhibit a Heaviside-like function variation with the wavelength and a wavelength cut-off at the vicinity of λ_cut-off_ ~ 400 nm. Above such a wavelength, the diffuse reflectance reaches promptly a plateau in the VIS and NIR spectral regions. This Heaviside function-like variation is close comparable to a perfect selectivity response as represented by the dashed yellow curve in Fig. 5a. Higher is the annealing temperature, higher is the plateau. The average value of the plateau of Reflectivity varies from 45 to 63% for samples annealed within 300–700 °C temperature range with a significantly low reflectivity in the UV-Bleu i.e. below λ_cut-off_ ~ 400 nm. In this UV spectral region, the reflectivity exhibits a significant fluctuation with an average of 0.7%. Because of the opacity of the CeO_2_ pellet and low reflectivity in this range, the UV selectivity is likely to be absorption dominated. This UV absorption is likely to be governed mainly by the transition of O 2p2 − to Ce 4f. 4 + as schematically represented in Fig. 5b. The optical transmission of thin pellets (thickness < 0.1 mm) of the various samples is reported in Figure S.2). As one can notice, the optical transmission is significantly low within the limit of detection both in the UV and bleu spectral regions. It can safely be deduced the absorption in the UV spectral region A = 1-R-T is dominant (A ~ 100%).

### ROS activity and photostability studies

In relation to the UV ROS photostability, Fig. 5c displays the relative Reactive Oxygen Species (ROS) production versus concentration of the biosynthesized nano-CeO_2_ as well as that of standard nano-TiO_2_ used as a reference for comparison. While the ROS production of nano-TiO_2_ increases significantly with the concentration reflecting its elevated photoactivity. In contrast, the ROS production of the biosynthesized nano-CeO_2_ seems stabilizing at very low values over the investigated concentration range of 0.5–1000 μg/ml. This high photostability is, likely, to be correlated to the high stoichiometry of the bio-synthesized CeO_2_ and the Ce4 + dominant valency in agreement with the XPS studies. Any non-stoichiometry in CeO_2_ arising from O deficiency both surface or in volume would be responsible for the production of free charge carriers upon UV irradiation and hence a ROS increase. The faster recombination rate of any generated e–h pairs in CeO_2_ relatively to that of TiO_2_ could explain such a low rate of ROS production^9,11,12^. In addition, there is a need to investigate the photostability as this parameter is of a pivotal role^19,20^. Accordingly, photostability studies were conducted using standard UV illuminations with long exposure time of 120 min. For such, a Solar Light’s advanced Model 601 Multiport® SPF Testing Solar Simulator was used. It is the industry standard for high throughput SPF testing and dermatological studies. It produces UVA or UVA + B (290–400 nm)/300 W. The samples were exposed to UVA + B during 120 min each with a 2 cm beam spot. The diffuse reflectivity was measured before and after such UVA + B irradiations. Figure S.3 reports the reflectivity of the 300 °C and 700 °C annealed samples before and after the UVA + B irradiations. As one can notice, and excluding the statistical fluctuations, there is nearly no significant change in the reflectivity profiles. Consequentially, it is safe conclude on the UVA + B photostability of the biosynthesized nano-CeO_2_. Such a photostability could be linked to the dominant Ce4 + valence and/or absence of oxygen deficiencies. Nonetheless, it is necessary to reconduct such a study once the nano CeO_2_ nanoparticles are embedded in a standard formulation for real life cosmetic applications.

## Conclusion

In summary, this study validated the efficiency of Hoodia *gordonii* natural extract as an effective agent for the biosynthesis of single phase CeO_2_ nanocrystals. Such bio-engineered nanocrystals exhibited a remarkable UV filtering optical selectivity quasi-similar to a perfect UV filtering system. In view of their negligible reactive oxygen species production, such a UV selectivity is conjugated to an exceptional photostability (Relative ROS < 0.5). Relatively to ordinary nanoscaled TiO_2_ and ZnO used in standards cosmetics, The currently bio-engineered CeO_2_ exhibited a superior photostability correlated to a low formation of harmful reactive intermediates such as singlet oxygen and reactive oxygen species (ROS) including Hydroxyl radicals which not only damage the DNA plasmids but also the skin cells. in support of their potential application novel green UV protective nano-cosmetics specifically, and skin protection in general. Nonetheless, it is necessary to reconduct such a study once the nano CeO_2_ nanoparticles are embedded in a standard formulation for real life cosmetic applications.

## Supplementary Information

Supplementary Information.
