# Supplementary material for: From Khoi-San indigenous knowledge to bioengineered CeO2 nanocrystals to exceptional UV-blocking green nanocosmetics
Source: Sci Rep. 2022 Mar 2;12:3468. doi: 10.1038/s41598-022-06828-x (PMC8891367; doi:10.1038/s41598-022-06828-x)
Supplement: Supplementary file 1 — Supplementary Information. [file 41598_2022_6828_MOESM1_ESM.pdf]

ADDITIONAL INFORMATION

From Khoi-San Indigenous Knowledge to Bioengineered CeO_2 Nanocrystals to exceptional UV-Blocking Green Nanocosmetics

S2.3. Surface& Interface analysis: XPS studies

Likewise, the XPS studies of the annealed CeO_2 nanoparticles were performed to investigate precisely the oxidation state of Ce and O (Fig. S1). The binding energy of CeO_2 nanoparticles exhibited several bands with the main ones centered at about 882.2, 888.5, 897.7 and 916.3 eV corresponding to the Ce^{4+} 3d3/2 and Ce^{4+} 3d5/2 in addition to the O1s peak at 531 eV. The XPS observations are consistent with the reported values in the literature (14-18). Hence, one can conclude that Ce is likely to be in 4+ valence state.

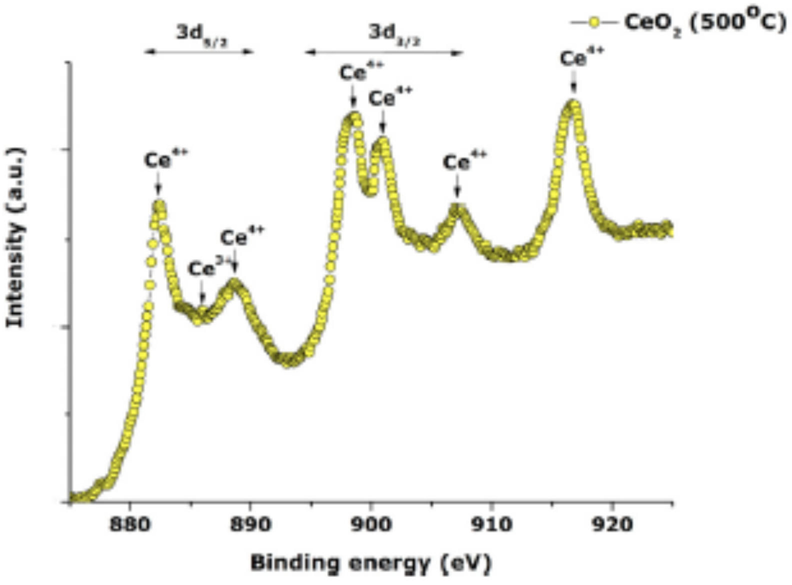

Fig. S1: Representative XPS spectrum of CeO_2 nano-powder annealed at ~700 °C .

S2.4. UV Optical selectivity

As it was mentioned previously the diffuse reflectivity is significantly low in the UV-Bleu i.e. below $\lambda_{cut-off}$ ~400 nm. In this UV spectral region, the reflectivity exhibits a significant fluctuation with an average of 0.7%. The optical transmission of thin pellets (thickness ~ 0.1 mm) of the various samples is reported in Figure S2). As one can notice, the optical transmission is significantly low within the limit of detection both in the UV & bleu spectral regions except for the 700 °C annealed sample (yellow triangles). It can safely be deduced the absorption in the UV spectral region A=1-R-T is dominant (A~100%).

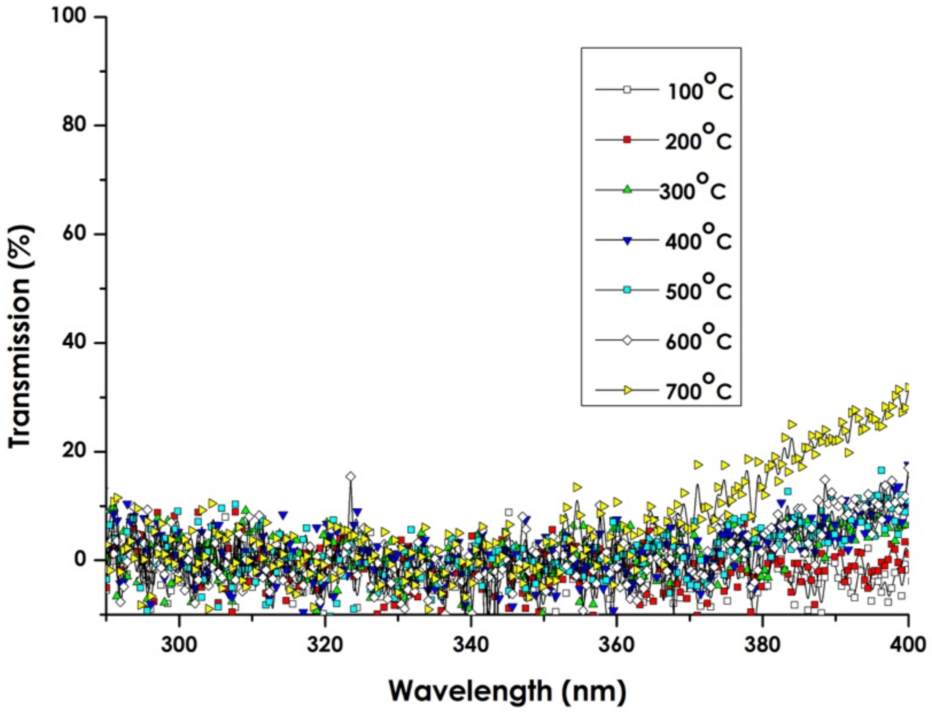

Fig. S2 : Optical transmission of the various CeO_2 pellets (~ 1mm in thickness) in the spectral range of 290-400 nm.

S2.5. ROS activity & photostability studies:

There is a need to investigate the photostability as this parameter is of a pivotal role (19-20). Accordingly, photostability studies were conducted using standard UV illuminations with long exposure time of 120 min. For such, a Solar Light's advanced Model 601 Multiport® SPF Testing Solar Simulator was used. It is the industry standard for high throughput SPF testing and dermatological studies. It produces UVA or UVA+B (290-400nm)/300W. The samples were exposed to UVA+B during 120 min each with a 2 cm beam spot. The diffuse reflectivity was measured before and after such UVA+B irradiations. Figure S3 reports the reflectivity of the 300°C& 700°C annealed samples before & after the UVA+B irradiations during 2 hours time. As one can notice in Fig.S6.b, and excluding the statistical fluctuations, there is nearly no significant change in the reflectivity profiles. Consequentially, it is safe concluding on the UVA+B photostability of the biosynthesized nano-CeO_2. Such a photostability could be linked to the dominant Ce4+ valence and/or absence of oxygen deficiencies. Nonetheless, it is intended to reconduct such a photostability once the nano CeO_2 nanoparticles are embedded in a standard formulation for real life cosmetic applications.

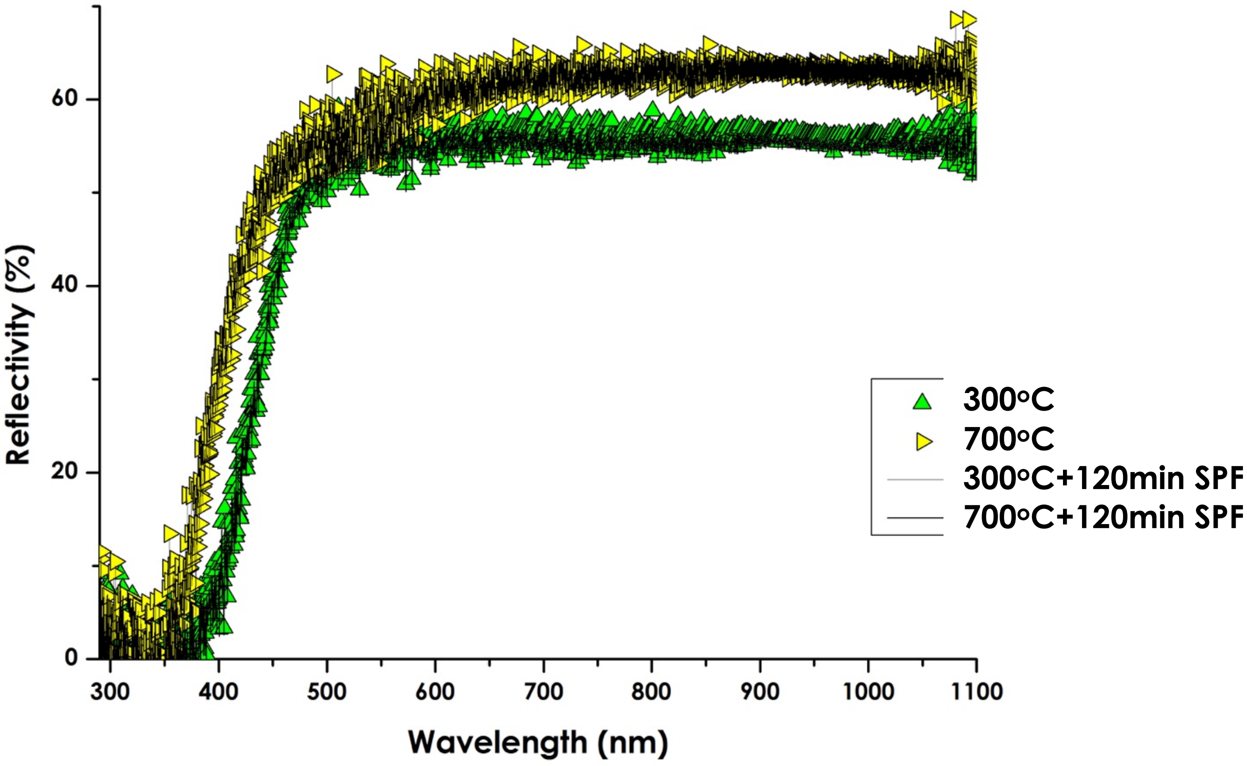

Fig. S3 : (a)Diffuse Reflectivity of the CeO_2 samples annealed at 300 & 700°C (green & yellow triangles) without SPF exposure and after 120 min SPF exposure (Continous lines).

S2.6. Size measurements studies:

The size analysis of the CeO_2 nanoparticles were estimated via various approaches; J-image data analysis of the TEM images (such as those of Fig.3), through the XRD profiles via the Scherrer approximation $<\phi> = (\lambda/\mathrm{Sin}\Theta_B\Delta\Theta_{1/2})$(from Fig3.f) as well as from Dynamic Light Scattering (DLS) investigations. This latter were carried at room temperature on ultrasonicated suspensions of the Ceria nanoparticles in water using a Nanotrac Flex DLS unit with a capability of measuring the nanoparticles' size within the range of 0.3 nm-15 μm (as per the technical specifications of the supplier) via the Dynamic diffusion coefficient approximation ($D=k_BT/3\ \pi\eta\ \phi$). The corresponding size distributions are reported in Fig.3.e.
